# A rare clinical image of hypertrichosis (Werewolf syndrome)

**DOI:** 10.11604/pamj.2022.43.99.37150

**Published:** 2022-10-25

**Authors:** Twinkle Joshi, Vaishali Kuchewar

**Affiliations:** 1 Department of Kayachikitsa, Mahatma Gandhi Ayurveda College, Hospital and Research Centre, Salod (H), Datta Meghe Institute of Medical Sciences (DU), Sawangi, Wardha, India

**Keywords:** Hypertrichosis, congenital, acquired

## Image in medicine

Hypertrichosis refers to enormous hair growth over the body. It is a rare condition in which the hair can cover the entire face and body or can occur in small patches. It can affect both men and women. There are several types of acquired hypertrichosis, nevoid hypertrichosis, congenital hypertrichosis lanuginosa, congenital hyertrichosis terminalis and hirsutism. These disorders can have either vellus, lanugo or terminal type of hair. There has not been any specific etiology, but it could be linked to porphyria cutanea tarda, androgenic steroids, lichen simplex, or malnutrition. Also, medications such as minoxidil, phenytoin, and cyclosporin have been linked to abnormal hair growth. Since there is no specific treatment, it can only be managed through epilation, shaving, electrolysis, or laser surgery. A 10-year-old male child approached with a patch of dark hair over the face that was present since birth. His clinical and medical history was not significant. On examination, the hair was dense, and thick and indicated a terminal type of growth. The child had low self-esteem and was also hesitant to talk about the problem. Since there have been very few cases around the globe there is limited research data for the treatment of this disorder it becomes necessary to guide such patients and boost their morals so that they could spend their lives like normal human beings.

**Figure 1:**
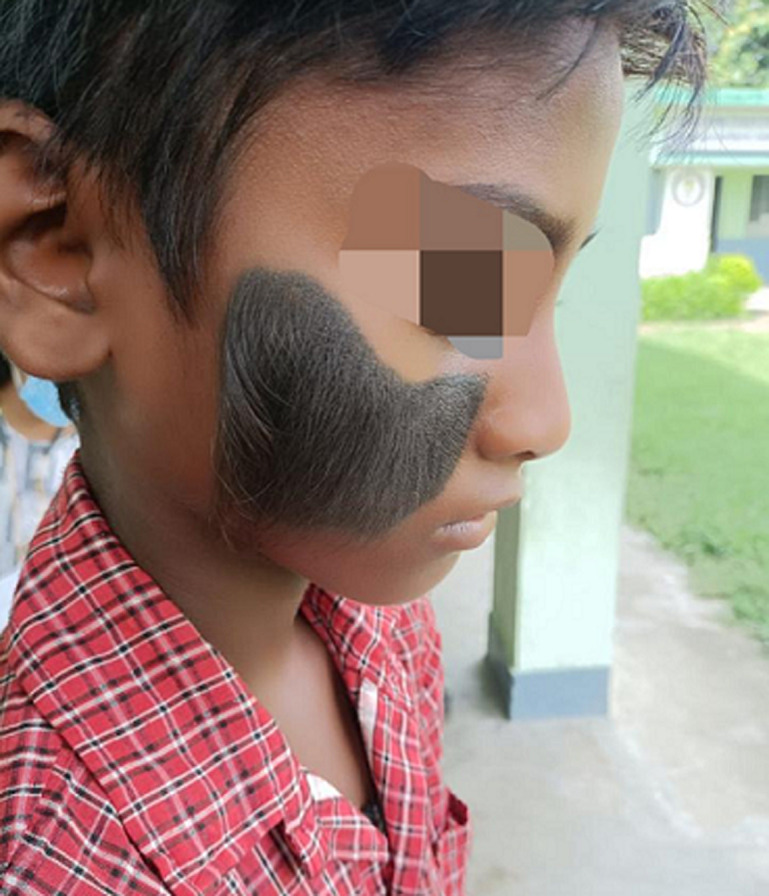
congenital hypertrichosis terminalis

